# Suppression of olfactory signal transduction by insecticides

**DOI:** 10.1038/s41538-019-0042-z

**Published:** 2019-06-03

**Authors:** Hiroko Takeuchi, Takashi Kurahashi

**Affiliations:** Department of Biophysical Dynamics, Graduate School of Frontier Biosciences, Osaka University, Suita, Osaka 5658701 Japan

**Keywords:** Cyclic nucleotide-gated cation channels, Inhibition, Olfactory receptors

## Abstract

2,4,6-Trichloroanisole (TCA) is a well-known, potent off-flavour compound present in various foods and beverages. TCA has been hypothesised to be a universal cause of flavour loss experienced in daily life. Here, however, we show that titres for the suppression of olfactory transducer channels caused by low-quality bananas are much higher than those for that caused by the TCA itself contained in the banana. We resurveyed other components of low-quality bananas and found that bananas also contain an insecticide (chlorpyrifos), and that it suppresses olfactory transducer channels. Other insecticides also suppressed olfactory transducer channels. Hence, even after passing safety examinations, certain insecticides may decrease the quality of foods and beverages by reducing their intrinsic scents.

## Introduction

In the food and beverage industry, various volatile compounds, called as off-flavour compounds, are known to potently degrade the flavour of products. Off-flavours are usually developed by chemical reactions in foods and beverages. In addition, non-preferred volatiles invading from outside belong to off-flavours. One of the most potent off-flavour compounds identified till date is 2,4,6-trichloroanisole (TCA), which has been associated with cork taint in wines.^1–4^ Human olfactory sense can recognise TCA, even at low concentrations of parts per thousand, from a wide variety of products.^5^ Therefore, TCA in foods and beverages has been hypothesised to be a universal cause of flavour loss in our daily life. Here, however, we show that titres for the suppression of olfactory transducer channels caused by the extracted vapour from low-quality bananas were higher than those caused by the TCA itself contained in bananas. These data indicate that bananas contain potent suppressors of olfactory transducer channels other than TCA. While examining the composition of volatile compounds emitted from bananas for TCA-related compounds, we found that bananas comprised the widely known insecticide, chlorpyrifos. Chlorpyrifos and other insecticides were experimentally confirmed as potent suppressors of olfactory transducer channels. These results suggest that certain types of insecticides, even after passing safety examinations, suppress olfactory transduction and reduce pleasant flavours of such foods.

## Results

### Single-cell responses to banana types

In 2013, Takeuchi et al.^6^ demonstrated that low-commercial value bananas contain relatively high concentrations (1500 ppt in banana peel) of TCA and that TCA suppresses cyclic nucleotide-gated (CNG) channel currents even at sub-femto molar ranges. Based on these findings, we examined the effects of high- and low-commercial value bananas on single olfactory receptor cell (ORC) excitation.

**Fig. 1:**
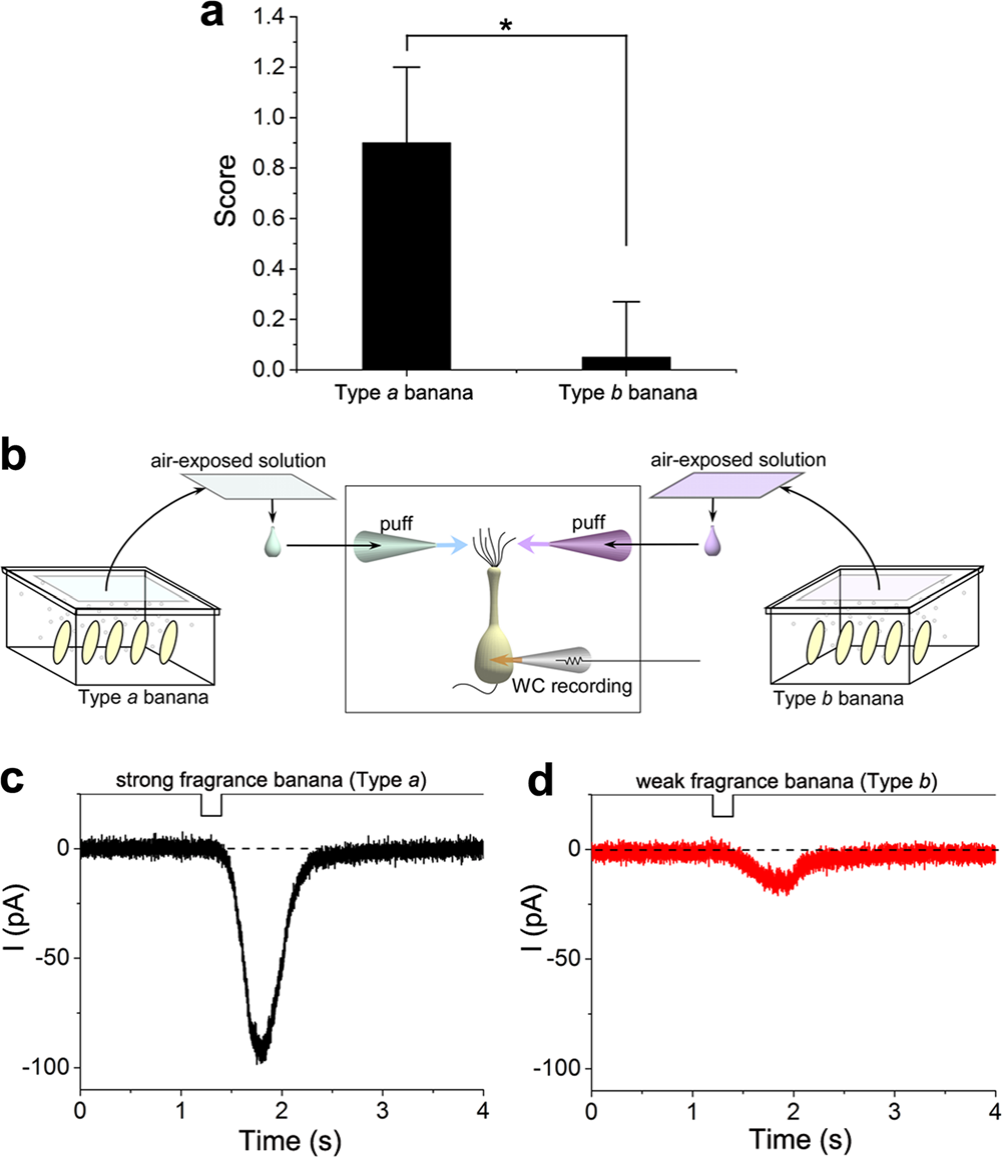
Cell responses to vapours from bananas. **a** Evaluation of banana flavour. Forty evaluators choose the stronger banana-like scent one from two bananas (paired test). For analyses, the score for selected banana was set to 1 and another 0. When the evaluator selected neither, both were 0. There is a significant statistical difference by Welch’s *t* test (*p < 0.05). **b** Scheme of cell stimulation; two puffer pipettes contained stimulants originated from type *a* or type *b* bananas. Sliced bananas were placed in containers for 3 h and volatile molecules in containers were adsorbed into filter papers that were wetted with normal Ringer’s solution and were adhered to the back of the lid. Solutions were then squeezed and applied to single olfactory receptor cells (ORCs) under the voltage clamp. **c** Current responses to stimulants from strong smelling bananas (type *a*). **d** Current responses to stimulants from weak smelling bananas (type *b*). **b**, **c** were obtained from the same ORC. The same banana samples (type *a* and *b*) were used for human psychology tests and cell experiments throughout **a**–**d**

For the study, two types of bananas, type *a* and *b* were purchased from the supermarket. These types predominantly differed regarding their market prices (type *a* had a higher value than type *b* bananas). Subsequently, 40 healthy evaluators judged which bananas had stronger banana scents only by sniffing. Among these, 36 identified type *a* as stronger, 2 identified type *b* as stronger and 2 could not distinguish between the types. When the score of chosen banana was set to 1 and another 0, there was a significant statistical difference between type *a* and *b* (Fig. 1a). The significance was large enough, even though the evaluators were non-experts regarding sensory tests. These data indicate that type *a* bananas (referred to as ‘high quality’) have stronger banana scents, as reflected by the commercial value.

Next, vapours from individual bananas were collected as described in Fig. 1b. These vapours from both banana types adsorbed onto Ringer’s solution-containing filter papers were then puff applied to 15 single ORCs under the whole-cell recording configuration using double puffer pipettes. Of these, five cells showed an inward current response to vapours from both banana types and the remaining 10 cells showed no remarkable responses. Of the 19 cells that were exposed to stimulants from type *a* bananas, only one cell exhibited a response. In addition, 17 cells were exposed to stimulants from type *b* bananas and only one exhibited a response. The responding cells were assumed to have receptor proteins that bind to some components of banana vapours. For five cells that exhibited responses to both types, amplitudes of inward currents were always higher following the exposure to vapours from type *a* bananas than those following the exposure to vapours from type *b* bananas (Fig. 1c, d) with average amplitudes of 130.0 ± 62.4 pA (mean ± SD, *n* = 5) and 52.9 ± 59.1 pA, respectively. This difference in response amplitudes may be caused by (i) the presence of a suppressor of olfactory transducer channels in type *b* bananas or (ii) a reduction in intrinsic compounds in type *b* bananas. These possibilities are examined in the following section.

### Suppression of cell responses by type *b* bananas

**Fig. 2:**
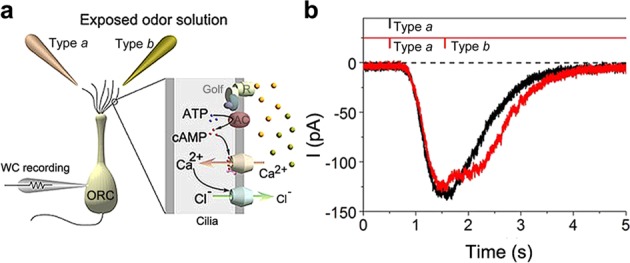
Cell response was suppressed by vapours from bananas. **a** Experimental scheme with a cytoplasmic molecular cascade. **b** Single pulse responses to the stimulant from type *a* bananas. Exposed-odour solution from type *a* banana induced an inward current (black). Current response to a double pulse stimulation (red). Note that the stimulant from type *b* banana suppressed the response to that from type *a* banana

**Fig. 3:**
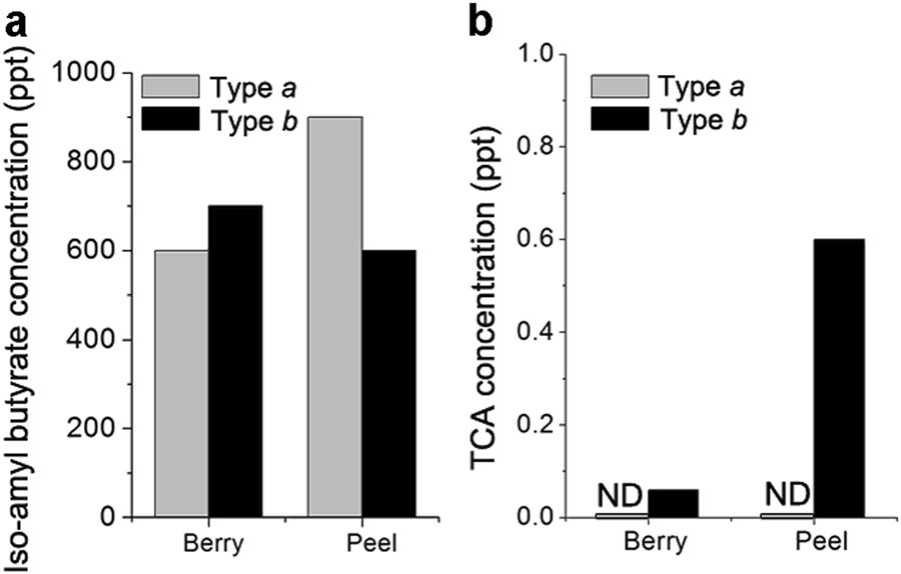
Analysis of fragrance components from bananas using GC/MS. **a** Concentrations of iso-amyl butyrate in type *a* and *b* bananas. **b** Concentrations of 2,4,6-trichloroanisole (TCA) in type *a* and *b* bananas; ND, not detected

Because smaller responses to vapours from type *b* bananas may be due to intrinsic suppressors, we determined the presence of suppressors in vapours emitted from type *b* bananas (Fig. 2a). When stimulants from type *b* bananas were applied to the peak of responses induced by stimulants form type *a* bananas, the amplitude of inward current significantly reduced (Fig. 2b), suggesting that vapours from type *b* bananas contain suppressors of olfactory transducer machinery. Alternatively, smaller responses to stimulants from type *b* vapours may reflect reduced amounts of intrinsic volatile compounds. We checked gas chromatography/mass spectrometry (GC/MS) data to confirm the chemical contents including in each banana (Fig. 3). However, concentrations of iso-amyl butyrate, a representative source of banana flavour, were not differed between type *a* and *b* bananas (Fig. 3a). These results indicated that smaller responses to stimulants from type *b* bananas were due to the presence of suppressors; this justifies why a majority of evaluators reported type *a* bananas to have the stronger banana scents.

**Fig. 4:**
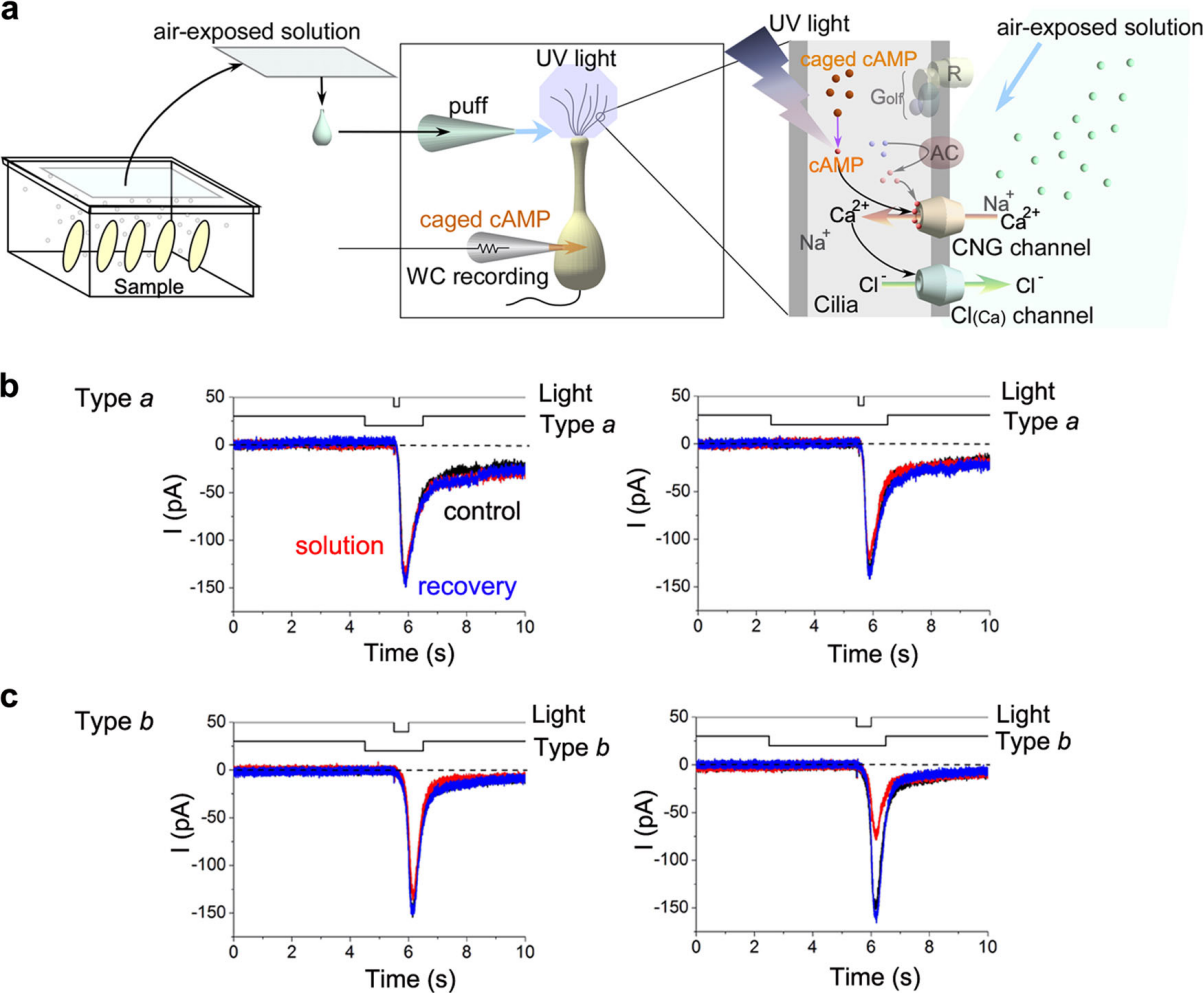
Vapours suppress cAMP-induced currents. **a** Scheme of protocol. **b** Current suppression by type *a*; stimulus solution was applied at 1 s (left) and 3 s (right) before light stimulation. Black trace (almost completely hidden by other traces) shows a current response to a cytoplasmic cAMP. Red trace shows the same response during a puff application of air-exposed solution from the banana *a* samples. Blue trace shows a recovery. Stimulants were applied at 1 s (left) and 3 s (right) before the light stimulation. The cell showed no response to banana vapours. **c** Current suppression by type *b* compounds. Colours of traces, control = black, red = stimulants, blue = recovery, were used throughout the text

Since iso-amyl butyrate is not an only component that determines the scent of the banana, it was possible that the concentration of other substances was reduced in type *b* banana. Therefore, next, we tried to identify the precise suppressive mechanisms underlying second messenger-mediated olfactory signal transduction with single ORC recordings and the photolysis of caged cAMP in the cilia (Fig. 4a). When stimulants from type *a* bananas were applied 1 s prior to the increase in cytoplasmic cAMP, cAMP-induced responses were not remarkably affected (Fig. 4b), whereas stimulants from type *b* bananas showed significantly reduced cAMP-induced responses (Fig. 4c). However, after a 3 s stimulus period, stimulants from type *a* bananas induced small reductions in cAMP-induced responses (Fig. 4b) and those from type *b* further enhanced the reduction in these responses (Fig. 4c). Similar time integrations of seconds were previously reported for determining suppressive effects of TCA that causes suppression of the CNG channel, suggesting that the suppression is caused downstream of adenylyl cyclase, most likely at the CNG channel.

### Comparison with TCA titration curves

TCA is a well-known, potent off-flavour compound and a strong suppressor of olfactory transducer channels.^6^ Furthermore, TCA has been found in a variety of products, particularly those of low quality and low-commercial value.^6^ Herein, TCA was only detected in vapours of type *b* bananas (Fig. 3b). Therefore, the suppressor present in banana vapours is, at least in part, likely to be TCA. Small reductions in responses following long exposures to vapours from type *a* bananas most likely reflect suppressive effects of various substances originally included in bananas. Among these, *n*-amyl acetate is a well-known major component of banana flavour and can suppress cAMP-induced responses of olfactory cilia.^7,8^

To verify the possibility of TCA suppression, we quantitatively investigated suppressive effects of banana vapours with reference to a TCA titration curve. We assumed that TCA concentrations are extremely low in extracts, and, therefore, the original sample solution was concentrated for measurements.

**Fig. 5:**
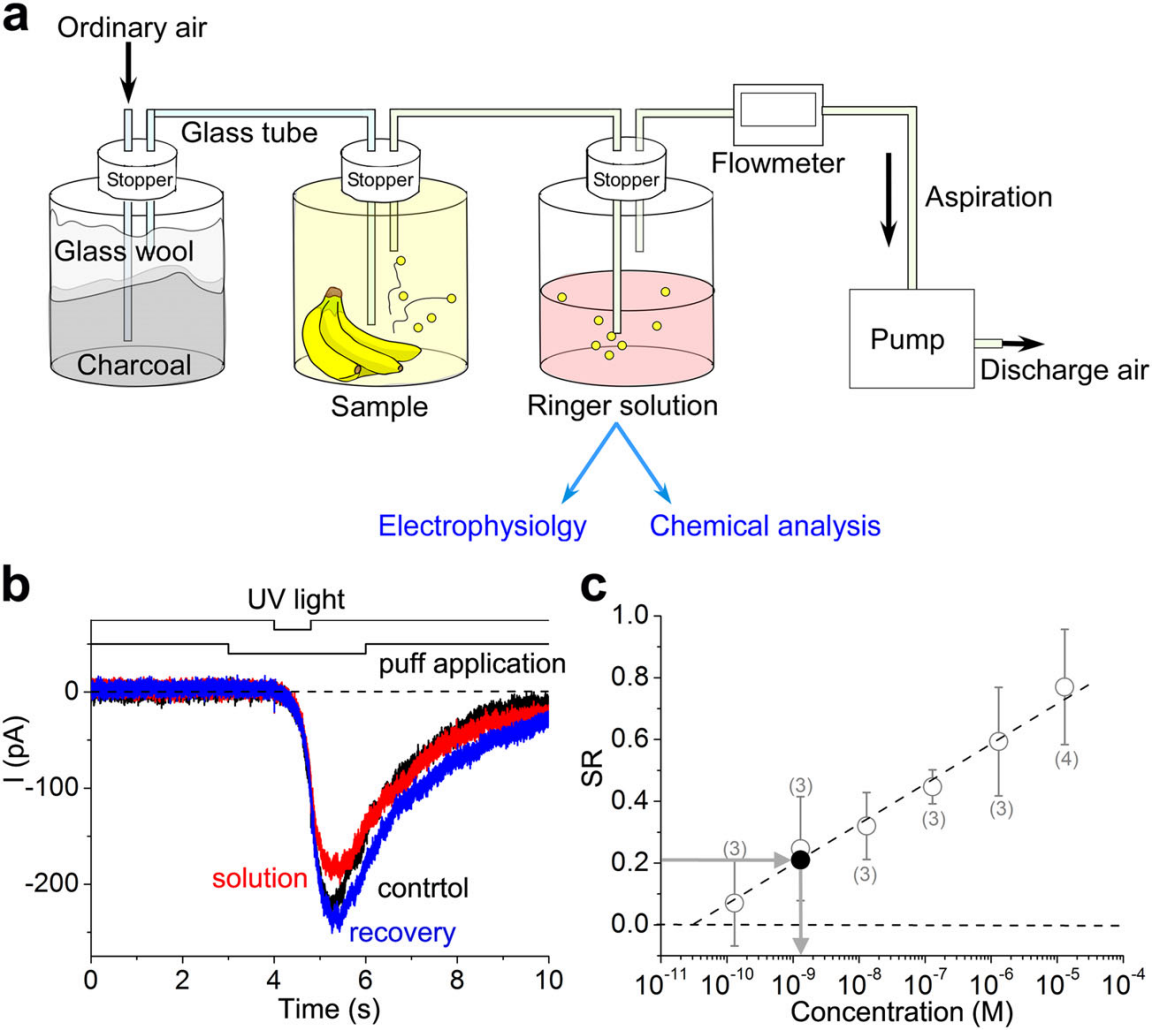
Estimation of 2,4,6-trichloroanisole (TCA). **a** Scheme showing procedures for the collection of volatile components; bubbling time, 17 h. **b** Effect of Ringer’s solution bubbled with vapours from type *b* bananas; puff duration, 3 s until 1 s before light stimulation; suppression ratio = 0.21. A cell showing no response to banana vapours; pressure 50 kPa. **c** *E*stimation of TCA concentrations; the calibration curve was obtained from a previous work^6^ (white circles). Estimated TCA concentration from the degree of suppression was 1.3 nM (black full circle)

In the experiments shown in Fig. 1, however, only small amounts of Ringer’s solution were exposed to vapours; these conditions may not be appropriate for quantitative assessments of suppressive effects of the volatile compounds. Thus, a large volume of Ringer’s solution (200 ml) was bubbled with vapours from type *b* bananas for 17 h (Fig. 5a), and the solution was simultaneously used in chemical analyses and cell-based assays.

Figure 5b shows the effect of Ringer’s solution after bubbling with vapours from type *b* bananas on cAMP-induced responses. As expected, cAMP-induced responses were suppressed by 1 s pre-applications of the stimulus solution. The concentration of suppressors in these experiments was thought to be lower than that in the experiments shown in Fig. 1. Furthermore, 1 s exposures to vapours from type *a* bananas had little effect on cAMP-induced responses, even using the protocol used in Fig. 1. Therefore, the suppression observed in these experiments (Fig. 5b) was mainly caused by a substance exclusively present in type *b* bananas. The degree of suppression (suppression ratio, SR) was 0.18 ± 0.02 in the three examined cells and was equivalent to that of 1 nM TCA when estimated from dose–suppression curves obtained under same experimental conditions (Fig. 5c, see also ref. ^6^).

Simultaneously, TCA concentrations in the same solutions were analysed using GC/MS. TCA was detected in the concentrated solution, and the TCA concentration in the original solution was extrapolated to be 0.5 pM, with some original solutions having a concentration 2000-fold lower than 1 nM. This large difference in concentrations strongly indicates that the bubbling of vapour incorporated CNG channel suppressors other than TCA in the vapour. Hence, the reduction of scent in type *b* bananas may primarily be due to unknown substances not present in type *a* bananas.

**Fig. 6:**
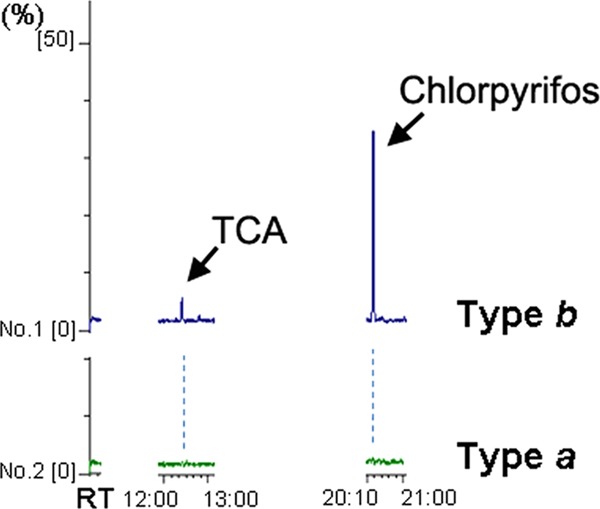
Measurements of components from type *a* and *b* bananas using GC/MS. Left column shows 2,4,6-trichloroanisole (TCA), right column chlorpyrifos in type *a* and *b* banana. The bananas were once frozen at −20 °C for stock. *Y*-axis shows relative percentage, and *X*-axis the retention time (RT)

We reanalysed volatile compounds in type *b* bananas using GC/MS and compared these with those from type *a* bananas. Because one of the remarkable differences between type *a* and *b* bananas is the presence of TCA and because TCA is a potent CNG-channel suppressor, we focused on components with chemical structures similar to that of TCA. These analyses identified chlorpyrifos only in vapours from type *b* bananas (Fig. 6). We then confirmed that chlorpyrifos has three Cl residues; moreover, it is commonly used as an insecticide in a wide variety of products, including bananas.

### Inward current suppression by chlorpyrifos

**Fig. 7:**
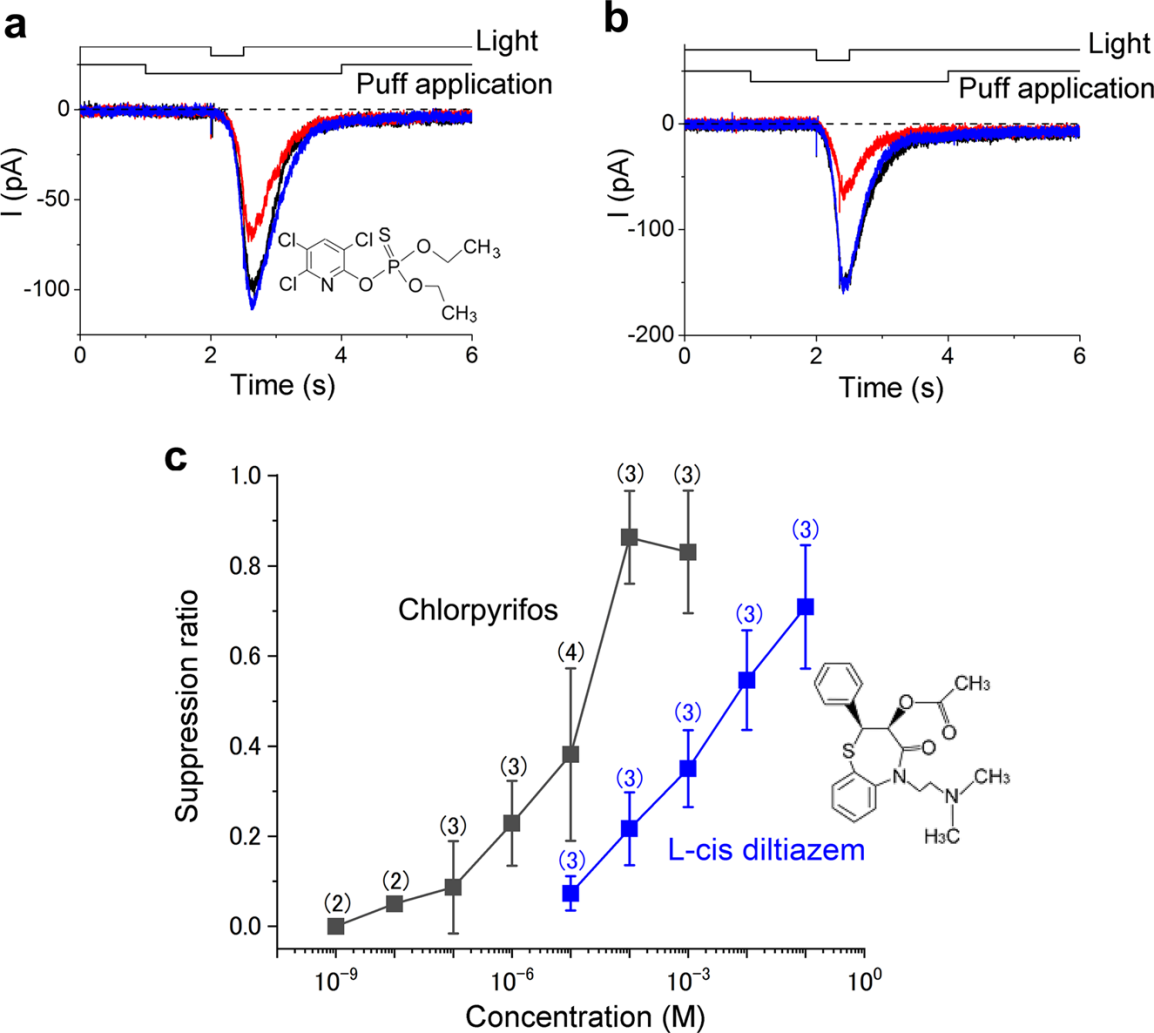
Comparison of current suppression by chlorpyrifos and l-*cis* diltiazem. **a** Current suppression by 1 μM chlorpyrifos; suppression ratio (SR) = 0.29. **b** Current suppression by 10 μM chlorpyrifos; SR = 0.48. **c** Dose–suppression relationships of chlorpyrifos (black full squares) and l-*cis* diltiazem (blue full squares, data from ref. ^6^)

In further studies, we examined the effect of chlorpyrifos on cAMP-induced responses of single ORCs. After confirming cAMP-induced responses in ORCs, we treated cells with 1 μM chlorpyrifos and then exposed them to ultraviolet (UV) light, which photolyses cytoplasmic caged cAMP (Fig. 7a). Amplitudes of cAMP-induced responses were diminished by chlorpyrifos in the stimulant, but after the washout, response curves were almost completely recovered. The degree of suppression increased when the concentration of chlorpyrifos was increased to 10 μM (Fig. 7b).

SRs were plotted against the concentrations of chlorpyrifos, which demonstrated a monotonous increase (Fig. 7c). When compared with the suppressive effects of l-*cis* diltiazem,^6^ a well-known commercial CNG channel blocker, those of chlorpyrifos were higher.

### Suppression by other insecticides

**Fig. 8:**
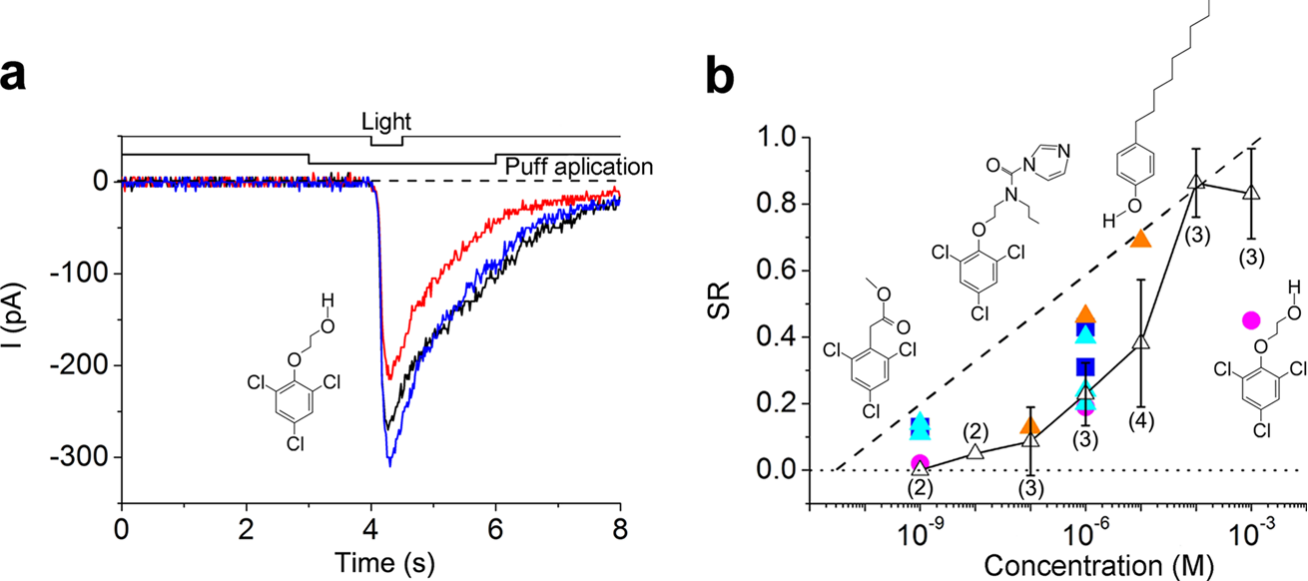
Current suppression by chlorpyrifos and insecticides. **a**, Current suppression by 1-μM 2,4,6-TPE (Log *D* = 3.36) SR = 0.19. **b** Dose–suppression relationship; blue full squares, prochloraz (Log *D* = 4.14, 3 cells); light blue full triangles, 2,4,6-TPA (Log *D* = 3.86, 5 cells); Pink full circles, 2,4,6-TPE (Log *D* = 3.36, 3 cells); orange full triangles, 4-nonylphenol (Log *D* = 6.13, 3 cells). White triangles, chlorpyrifos (Log *D* = 4.78); data were obtained from those reported in Fig. 5c. Dotted lines show the 2,4,6-TCA) calibration curve^6^

We also determined whether other insecticides have similar suppressive effects on olfactory transducer channels. We selected the following three insecticides with three Cl residues: 2,4,6-trichlorophenoxy ethanol (2,4,6-TPE; Fig. 8a), prochloraz and 2,4,6-trichlorophenylacetate (2,4,6-TPA). All tested chemicals markedly suppressed cAMP-induced responses in ORCs (Fig. 8b).

We performed further experiments using 4-nonylphenol as it lacks Cl residues and is a well-known off-flavour that has been detected in drains and is thought to be an environmental hormone. Surprisingly, 4-nonylphenol suppressed CNG channels with almost the same effective concentration as the Cl-containing insecticides. It has been shown that the suppression of CNG channels by various volatile substances is dependent on water/oil partition coefficients at a constant pH (Log *D*). Notably, these compounds have similar Log *D* ranges.

## Discussion

In the present study, we show that certain types of insecticides suppress olfactory transducer channels. Accumulated evidence indicates that the suppression of olfactory transducer channels leads to reduced or modified olfactory senses.^6,8^ Therefore, it is highly likely that insecticides change food and beverage qualities. Their use in agriculture has long been discussed.

Recently, wines containing pesticides were proposed to alter quality.^9^ It has been known that olfactory information directly projects to deep parts of the brain, like limbic system,^10,11^ bypassing recognition steps. Therefore, it remained controversial if psychophysical trials for evaluating effects of contaminants provided a solid answer. Herein, we show that insecticides suppress olfactory signal transduction in the receptor cell and may do so at concentrations below those required to produce a dominant smell, as shown for low concentrations of TCA.^6^ Because flavours of products are tightly linked with olfaction, the suppression of olfactory transducer channels is likely to devalue food products.

The suppression manners of insecticides found here resemble to those of TCA. Both showed exposure-time dependences; the degree of suppression increased during the exposure of the time range of several seconds. Also, suppression of CNG channels has been reported for a variety of substances.^8^ Within structurally similar substances, the degree of suppression is nearly proportional to their oil/water partition coefficient, especially at the same pH (Log *D*).^12^ Also in the present study, substances having similar Log *D* showed similar SRs. Because of the time and hydrophobicity dependence, it is hypothesised that the suppression effect is started from the integration of these suppressive substances into the lipid bilayer of the plasma membrane. The suppression effect by insecticides may share the same strategies.

The safety of pesticides and insecticides has previously been considered in terms of human health, and the ensuing doses are determined only as those for which no health risks are detectable. Comparatively few studies consider changes in flavour due to very low concentrations of pesticide and insecticides in foods. At the same time, professional traders always pay attention to the qualities of their products as they determine the commercial value of these products. It has long been believed that the degradation of food odours predominantly follows a decrease in original flavour components. In this study, we suggest that the product quality decreases by compounds that inhibit olfactory function, even at very low concentrations. As a proof of this concept, our experiments show that although one of the sources of original banana flavour, iso-amyl butyrate, did not differ between low- and high-quality bananas, additional flavour-affecting compounds were present at concentrations that can suppress olfactory transducer channels. These compounds were likely to be present in bananas as a consequence of pesticide use. It may be necessary to reconsider pesticide and insecticide use considering olfactory perceptions.

## Methods

### Bananas

Two types of fresh bananas, belonging to the Cavendish subgroup of the AAA cultivar group, were both purchased from the standard supermarket in the Osaka area of Japan. The most remarkable difference of bananas was their market prices, positioning both extremes in the store. The degree of ripeness of bananas would be uniform in Japanese supermarket. Indeed, their appearances were almost the same in yellow colours and shapes, and concentrations of the majour volatile included in both bananas were similar (see Results). The same raw bananas were, unless otherwise indicated, used for all of the quality evaluation, cell experiments and chemical analyses.

### Evaluation of banana scent

Evaluation of banana scents was conducted after informed consent was obtained orally in compliance with the guidelines for ethics regarding human epidemiological studies at Osaka University (approval no. FBS30-15). The volunteer evaluators, 8 female and 32 male undergraduate students in Osaka University, were unaware of the relative commercial values of the bananas. They had no prior sensory testing and the test did not use repeated trials. A complete double-blind procedure was established; neither the evaluators nor the technicians who presented samples were involved in other experiments or data analyses. Prior to the test, the evaluators learnt basics of sensory-physiology and neuro-physiology. At the test, the technician again confirmed the knowledge about the perception of odours and cell adaptation, and their molecular mechanisms.^13^ The evaluators were noticed to avoid olfactory fatigue/adaptation by taking inter-stimulus intervals. They received two types of bananas with the peel simultaneously, and sniffed one by one as they were repeatedly, until they identified the banana emitting stronger banana-like scents. The technician asked which banana has stronger banana-like scents, independent from personal preferences about the banana. Therefore, the evaluators must have selected the banana that emitted stronger banana-like scents, even if she/he did not like bananas personally. The activities were conducted in an air-conditioned room with a standard room light.

### Cell dissociation

ORCs were enzymatically dissociated from olfactory epithelia of newts (*Cynops pyrrhogaster*) as previously described, and were identified by their morphology and electrical properties.^12,14,15^ The animal was used because they have larger ORCs than other vertebrates. Experiments were performed in compliance with the latest ethical guidelines for animal experimentation at the Osaka University based on international experimental regulations. Animals were anaesthetised by cooling at 4 °C and then double-pithed. After decapitation, olfactory epithelia were removed and were incubated (37 °C, 5 min) in Ca^2+^- and Mg^2+^-free 0.1% collagenase solution containing 110 mM NaCl, 3.7 mM KCl, 10 mM HEPES, 15 mM glucose, 1 mM pyruvate and 0.001% phenol red (pH 7.4). Single cells were finally mechanically isolated. The resultant cells were then placed on concanavalin A-coated glass-bottom culture dishes and were washed using normal Ringer’s solution containing 110 mM NaCl, 3.7 mM KCl, 3 mM CaCl_2_, 1 mM MgCl_2_, 10 mM HEPES, 15 mM glucose, 1 mM pyruvate and 0.0005% phenol red. All experiments were performed at room temperature (23–25 °C).

### Electrophysiology

Ciliary currents were monitored using a whole-cell recording configuration.^16^ Throughout the experiments membrane potentials were held at −54 mV after corrected for by liquid junction potentials at the pipette tip (4 mV).^14^ Culture dishes were mounted on a Nikon TMD for diffuse UV photolysis. Patch pipettes (resistance, 10–15 MΩ) made of borosilicate tubing (outer diameter, 1.2 mm; World Precision Instruments)^6,8,15^ were used. The recording pipette was filled with a 119 mM CsCl solution.^14^ The pH of pipette solutions was adjusted to 7.4 using CsOH in 10 mM HEPES buffer. For cAMP experiments, 1 mM CaCl_2_, 5 mM EGTA and 1 mM caged cAMP were added. Normal Ringer’s solution (as described earlier) was used as a superfusate in all experiments. The recording pipette was connected to a patch-clamp amplifier (Axopatch 200B; MDS Analytical Technologies). Signals were low-pass filtered at 0.5kHz and were digitised using an A/D converter (sampling frequency, 1 kHz) connected to a personal computer operating on MS-DOS (PC9821, 80486CPU; NEC) or MS-Windows (xw8600 workstation; HP) with the pClamp 8 software (MDS Analytical Technologies). Simultaneously, signals were monitored on an oscilloscope. UV light, odorant stimulation and data acquisition were regulated by the same computer using a custom software. Data were analysed using a workstation computer and were plotted using the Microcal Origin 7.5 software (OriginLab). For curve plots, data were smoothed using 50 Hz averaging or data that were sampled at 1/16 kHz were used.

### Photolysis of caged compounds

Single ORCs were loaded with caged cAMP [adenosine 3-5-cyclic monophosphate, P1-(2-nitrophenyl) ethyl ester of cAMP; EMD]. Caged cAMP (100 mM) was initially dissolved in dimethyl sulfoxide and was frozen at −20 °C in complete darkness. Prior to the experiments, stock solutions were diluted into pipette solutions to obtain a final concentration of 1 mM before each experiment. For photolysis, UV light from a 100 W xenon arc lamp was directed onto the cilia of solitary cells using an epifluorescence attachment. Light stimuli were then applied at >20 s intervals to avoid response adaptation. The time and duration of light illumination were controlled using a magnetic shutter (Copal), and the light intensity was adjusted using a wedge filter controlled using a computer. The light source was mechanically isolated from the microscope to exclude the transmission of vibrations.^15^

### Chemical stimulation

The chemicals chlorpyrifos, TPE, prochloraz, 2,4,6-TPA and 4-nonylphenol were purchased from the Tokyo Chemical Industry. All Log *D* values were obtained from the ChemSpider database of the Royal Society of Chemistry on 21 March 2019. Test chemicals were dissolved in Ringer’s solution and were applied to cells using pressure ejection^17^ from a glass puffer pipette with a tip diameter of approximately 1 μm.

### GC/MS analyses

A 7890A GC system (Agilent Technologies) and a Jms Q1000GC MKII system (JEOL) were used as described before.^6^

## Data Availability

The data that support the findings of this study are available from the corresponding author upon reasonable request.

## References

[1] Buser HR, Zanier C, Tanner H (1982). Identification of 2,4,6-trichloroanisole as a potent compound causing cork taint in wine. J. Agric. Food Chem..

[2] Evans TJ, Butzke CE, Ebeler SE (1997). Analysis of 2,4,6-trichloroanisole in wines using solid-phase microextraction coupled to gas chromatography-mass spectrometry. J. Chromatogr. A.

[3] Taylor MK, Young TM, Butzke CE, Ebeler SE (2000). Supercritical fluid extraction of 2,4,6-trichloroanisole from cork stoppers. J. Agric. Food Chem..

[4] Alvarez-Rodriguez ML (2002). Cork taint of wines: role of the filamentous fungi isolated from cork in the formation of 2,4,6-trichloroanisole by o methylation of 2,4,6-trichlorophenol. Appl. Environ. Microbiol..

[5] Silva Teixeira CS, Silva Ferreira AC, Cerqueira NM (2016). Studying haloanisoles interaction with olfactory receptors. ACS Chem. Neurosci..

[6] Takeuchi H, Kato H, Kurahashi T (2013). 2,4,6-Trichloroanisole is a potent suppressor of olfactory signal transduction. Proc. Natl Acad. Sci. USA..

[7] Kurahashi T, Lowe G, Gold GH (1994). Suppression of odorant responses by odorants in olfactory receptor cells. Science.

[8] Takeuchi H, Ishida H, Hikichi S, Kurahashi T (2009). Mechanism of olfactory masking in the sensory cilia. J. Gen. Physiol..

[9] Séralini, G. E. & Douzelet, J. The Taste of Pesticides in Wines. *Food. Nutr. J.* FDNJ-161. 10.29011/2575-7091.100061 (2017).

[10] Lopes da Silva FH, Witter MP, Boeijinga PH, Lohman AHM (1990). Anatomic organization and physiology of the limbic cortex. Physiol. Rev..

[11] Ganong, W. F. Review of Medical Physiology: Smell and Taste. 21st edn. pp. 188–191 (McGraw Hill Companies Inc., New York, 2003).

[12] Kishino Y, Kato H, Kurahashi T, Takeuchi H (2011). Chemical structures of odorants that suppress ion channels in the olfactory receptor cell. J. Physiol. Sci..

[13] Kurahashi T, Menini A (1997). Mechanism of odorant adaptation in the olfactory receptor cell. Nature.

[14] Kurahashi T (1989). Activation by odorants of cation-selective conductance in the olfactory receptor cell isolated from the newt. J. Physiol..

[15] Takeuchi H, Kurahashi T (2002). Photolysis of caged cyclic AMP in the ciliary cytoplasm of the newt olfactory receptor cell. J. Physiol..

[16] Hamill OP, Marty A, Neher E, Sakmann B, Sigworth F (1981). Improved patch-clamp techniques forhigh-resolution current recording from cells and cell-free membrane patches. Pflüg. Arch.

[17] Ito Y, Kurahashi T, Kaneko A (1995). Pressure control instrumentation for drug stimulation. Nippon Seirigaku Zasshi.

